# Leveraging AI for Meta‐Analysis: Evaluating LLMs in Detecting Publication Bias for Next‐Generation Evidence Synthesis

**DOI:** 10.1002/cesm.70047

**Published:** 2025-09-18

**Authors:** Xing Xing, Lifeng Lin, Mohammad Hassan Murad, Jiayi Tong

**Affiliations:** ^1^ Department of Biostatistics Johns Hopkins Bloomberg School of Public Health Baltimore Maryland USA; ^2^ Department of Epidemiology and Biostatistics University of Arizona Tucson Arizona USA; ^3^ Evidence‐Based Practice Center, Mayo Clinic Rochester Minnesota USA

**Keywords:** evidence synthesis, large language models, meta‐analysis, publication bias

## Abstract

**Introduction:**

Publication bias (PB) threatens the validity of meta‐analyses by distorting effect size estimates, potentially leading to misleading conclusions. With advanced pattern recognition and multimodal capabilities, large language models (LLMs) may be able to evaluate PB and make the systematic review process more efficient.

**Methods:**

We evaluated the ability of two state‐of‐the‐art multimodal LLMs, GPT‐4o and Llama 3.2 Vision, to detect PB using funnel plots alone and in combination with quantitative inputs. We simulated meta‐analyses under varying conditions, including the absence of PB, different levels of presence of PB, varying total number of studies within a meta‐analysis, and differing degrees of between‐study heterogeneity.

**Results:**

Neither GPT‐4o nor Llama 3.2 Vision consistently detected the presence of PB across various settings. Under no‐publication‐bias conditions, GPT‐4o achieved a higher specificity outperforming Llama 3.2 Vision, with the difference most shown in the meta‐analyses with 20 or more studies. The inclusion of quantitative inputs alongside funnel plots did not significantly improve performance. Additionally, between‐study heterogeneity and patterns of non‐reported studies had minimal impact on the models’ assessments.

**Conclusions:**

The ability of LLMs to detect PB without fine‐tuning is limited at the present time. This study highlights the need for specialized model adaptation before LLMs can be effectively integrated into meta‐analysis workflows. Future research can focus on targeted refinements to enhance LLM performance and utility in evidence synthesis.

## 1 Introduction

Systematic reviews and meta‐analyses, which are at the top of the evidence pyramid for scientific evidence, are the cornerstone of identifying and appraising evidence for decision‐making in healthcare. However, they can be costly, take a long time to complete, and require large human effort. With the rapid advancements in large language models (LLMs), these tools are emerging as powerful assets in the evidence synthesis process. An explosion of the use of LLMs in systematic reviews has taken place since 2022. Starting with the first step in a systematic review, literature search [1], moving to study selection [2], data extraction [3, 4], and assessment of methodological quality of primary included studies [5, 6].

While these efforts have shown potential application for LLMs in systematic reviews, for example, tools like Semantic Scholar, Elicit, and GPT‐based query reformulation have been useful in refining search strategies by aiding Boolean logic validation and term expansion. However, performance varies and recall remains a challenge when coverage is incomplete [7]. Despite their growing role in various stages of systematic reviews, LLMs have not been widely explored for their potential contributions in the meta‐analysis stage, particularly in the evaluation of publication bias (PB).

PB can concur at every stage of the dissemination process, from investigators’ decision to submit a manuscript, through editorial and peer‐review judgments, to the final act of journal publication, whenever those decisions are driven more by the study's results than by its methodological quality [8, 9]. In the context of meta‐analysis, PB typically refers to the tendency for studies with statistically significant findings to be more likely to be published, while those with null or nonsignificant results remain unpublished. As a result, the body of available evidence could systematically become skewed in the presence of PB. When meta‐analyses are conducted on this biased subset, the pooled estimate can be distorted and inflated, diverging from the true effect [10, 11, 12]. Selective publication of positive outcomes skews the evidence, potentially leading to misguided clinical guidelines and policy decisions based on inaccurate estimation of the balance of benefits and harms [13, 14]. Therefore, detecting and correcting PB within the meta‐analysis workflow is essential to ensure the reliability and validity of the findings.

Although various methods have been developed for PB detection and correction [13], such as funnel plot, Egger's regression test, Peters’ test, and Copas’ method [9, 11, 15, 16], their proper application in clinical research and the interpretation of their results remain inconsistent. Due to the limited availability of statistical expertise or lack of understanding of the limitations of these methods, the process of PB detection and correction is frequently neither rigorously conducted nor adequately reported in clinical papers [13, 17, 18], raising concerns about the reliability of the findings. For instance, many meta‐analyses include funnel plots without performing any quantitative tests for PB, and others make incorrect conclusions based on a very small number of primary studies [12, 17, 19]. Despite requiring comparatively little manual effort relative to other procedures in systematic reviews and meta‐analyses, PB assessment is often omitted or misapplied. Surveys indicate that fewer than 15% of recent healthcare meta‐analyses perform a formal test on PB, and more than half apply asymmetry tests under conditions considered inappropriate by the statistical guidance [17]. Visual inspection alone on PB is also not reliable; empirical studies have shown that researchers perform no better than chance using funnel plots to detect PB [20, 21]. Therefore, an automated, guideline‐informed PB module could add value not by saving time, but by promoting best practices and reducing common analytic errors. To inform the development of such a tool, it is first essential to evaluate the capabilities of existing models.

The aim of this study is to evaluate the capabilities of multimodal LLMs in detecting PB in meta‐analysis. In particular, we focus on two state‐of‐the‐art multimodal models: GPT‐4o [22], developed by OpenAI, and Llama 3.2 Vision [23], developed by Meta, both of which can process visual and textual data. We assess their performance in PB detecting and assessing PB using: (1) visual inputs alone (i.e., funnel plots) and (2) a combination of visual inputs with quantitative metadata, including estimated effect sizes, and corresponding variances, to provide more detailed information for funnel plots as a sensitivity analysis. Through this evaluation, we aim to explore how these AI‐driven models can shape the next generation of evidence synthesis by enhancing the accuracy, efficiency, and reproducibility of PB detection. By leveraging advanced multimodal LLMs, we seek to pave the way for AI‐assisted meta‐analysis, ultimately driving more reliable and data‐driven decision‐making in research.

## 2 Method

### 2.1 Simulation Design

Because real‐world datasets rarely offer a reliable ground truth for PB [24], we turned to controlled simulation studies to evaluate the ability of GPT‐4o and Llama 3.2 Vision to detect PB.

First, we simulated meta‐analysis level data using a hierarchical two‐stage process., For each simulated meta‐analysis, we generated the study‐specific effect sizes ($y_{i}$) by $y_{i}∼N\left(\mu_{i}\;,s_{i}^{2}\right)$, where the${\;\mu }_{i}$'s are study‐specific true effect size and $s_{i}$'s are within‐study standard errors. The true effect sizes were $\mu_{i}∼N(\mu ,\tau^{2}).$ The true overall effect size was set to μ=0 to ensure the generalization of our study. To reflect heterogeneity in study precision, the within‐study standard errors $s_{i}$ were independently sampled from a uniform distribution: $s_{i}∼\mathrm{Uniform}\left(1,4\right).$ We also considered the potential impact of between‐study heterogeneity on PB detection by setting the between‐study variance $\tau^{2}$ to 0 and 1.

Second, we simulated meta‐analysis under the presence of PB. The presence of PB was simulated from two perspectives: bias based on p‐values and bias based on effect sizes. Specifically,

- **Scenario 1 (PB based on p‐values):** In this scenario, the probability of a study being published is assumed to depend on the p‐value of its estimated effect size. Studies with smaller, statistically significant p‐values are more likely to be published, while those with larger, nonsignificant p‐values have a lower chance of publication. A two‐sided α level of 0.05 was used to define statistical significance.
- **Scenario 2 (PB based on effect sizes)**: In this scenario, the probability of a study being published depends on the magnitude of the estimated effect sizes. Studies reporting larger effect sizes are more likely to be published, regardless of their statistical significance.

In addition to scenarios involving PB, we also simulated a setting without PB as a reference group for evaluating the LLMs’ false positive rates.

To capture a more realistic, evidence‐based perspective, we set the number of studies in each meta‐analysis to 15, 30, 50, and 75, following the designs of previous empirical studies [25, 26]. In each meta‐analysis, we generated a total of m+n studies (i.e., i=1,…,m+n). we ranked all studies by the p‐value (based on Scenario 1) or effect sizes (based on Scenario 2) and selectively removed *M* studies with less favorable results. Here, m represents the “unpublished” studies excluded under the two bias scenarios to reflect the presence of PB, while n represents the “published” studies used to create the funnel plot. Specifically, under different bias scenarios, we excluded studies with the smallest absolute effect sizes or those with negative or nonsignificant results to mimic the common patterns observed in empirical research, where studies with null or unfavorable findings are less likely to be published. The value of m was set to 0 (i.e., no PB), n/10, n/5, and n/3. These proportions describe how many of the studies were not published. Each simulation scenario was repeated 100 times.

Within the data‐generating process, some meta‐analyses could not be performed due to the non‐convergence of the Fisher scoring algorithm when using the restricted maximum‐likelihood (REML) method, which was our primary and preferred method for estimating between‐study variance. To address this computational issue and ensure the effectiveness of the evaluation, we regenerated the study‐level data according to the corresponding scenario group until each scenario generated 100 meta‐analyses.

Overall, we simulated four scenarios based on the number of studies, including 15, 30, 50, and 75 studies, to represent small, moderate, and relatively large meta‐analyses. Within each number of studies’ scenario, we reported the consistency rates across various PB settings, including severe PB (33% of studies excluded based on p‐value or effect size), moderate PB (20% excluded), mild PB (10% excluded), and no PB (no studies excluded, i.e., absence of PB).

### 2.2 LLMs

Within the scope of this study, we employed and compared two of the most widely used multimodal LLMs: GPT‐4o and Llama 3.2 Vision. GPT‐4o [22], developed by OpenAI, is a highly advanced language model known for its proficiency in both text and image understanding, making it well‐suited for analyzing complex inputs like funnel plots. Llama 3.2 Vision [23], developed by Meta, is a multimodal model capable of processing both visual inputs, such as images or plots, and textual data.

All statistical code and data used for implementing the methods are publicly available at https://osf.io/e2quz/. Analyses were conducted using R (version 4.3.1). For LLMs, GPT‐4o was accessed through OpenAI's platform, and Llama 3.2 Vision (11B parameters) was evaluated locally using an A100 40GB GPU for inference, provided by ARCH.

### 2.3 Input of LLMs

Our primary analysis is to evaluate the capabilities of multimodal LLMs to detect PB with visual input alone. With the simulated data, we generated the funnel plots (via the R package “*metafor*” [27] version 4.8‐0) as the primary visual inputs for the LLMs. Additionally, we aim to explore whether adding quantitative data alongside visual inputs can enhance LLM performance in PB detection. We also investigate whether providing more detailed information in funnel plots can improve inference. Therefore, we conducted a secondary analysis, supplying simulated study‐level data, including effect sizes and standard errors, as supplementary statistical inputs to further assess each LLM. After each simulation was completed, the funnel plot was rendered as a 600 × 600 px PNG image using ggplot2 [28]. The associated data (study‐level effect sizes, standard errors) were converted into JSON format. One image at a time was sent to the LLM. The main control script executed a for loop to: (i) read the PNG file and encode it in base64, and (ii) POST it to the API along with a JSON‐formatted study‐level data table.

### 2.4 Prompt Engineering

While our aim was to evaluate GPT‐4o and Llama 3.2 Vision for their ability to detect PB without fine‐tuning, these models still require appropriate prompts to effectively leverage contextual information and understand the task. Consequently, our methodology involves providing relevant context as part of the prompt to guide the LLMs’ reasoning and facilitate accurate assessment.

Specifically, within the primary analysis where PB was assessed using visual inputs only, we employed a language‐based prompt inspired by the Cochrane Handbook [29]. The prompts for funnel plots explain the key elements of a funnel plot about the axis and how it is used to assess PB. The specific prompt in Table 1 was used for engineering. In the secondary analysis, PB was assessed using combined visual and quantitative inputs. The prompt was built upon the previous one and aimed to introduce LLMs to leverage more information from statistical data. The following specific prompt in Table 1 was used for engineering based on the contextual prompt of plot‐based input. The output is Present/Absent of PB. To ensure stability and reproducibility of the LLM's outputs, we ran each prompt five times per funnel plot input. A correct identification was recorded only if all five outputs were exactly identical. This strict agreement criterion minimizes the influence of stochastic variation in LLM responses and ensures that only fully consistent outputs are accepted as valid model decisions.

**Table 1:** Contextual prompt information for LLMs.

Contextual statements	
Summary information about PB	A funnel plot is commonly used to assess publication bias in meta‐analyses. Each point on the funnel plot represents an individual study. The vertical axis (y‐axis) shows a measure of precision (e.g., standard error), and the horizontal axis (x‐axis) indicates the effect size. In the absence of publication bias, the funnel plot should resemble a symmetric inverted triangle. Asymmetry in the funnel plot may suggest publication bias.
Criteria to make the decision based on plots	Symmetry around the overall effect size—look for symmetry (minimal or no bias) or asymmetry with visible gaps (potential bias).
	Distribution of studies by precision—check if high‐precision studies cluster around the mean effect size, and if low‐precision studies are scattered symmetrically (balanced distribution = no bias, skewed clustering = potential bias).
	Effect size trends—identify if smaller studies show larger effects (no systematic change = minimal/no bias, clear trend = potential bias).
	Missing study patterns—look for gaps in expected regions, indicating selective publication (no gap = no bias, noticeable gaps = potential bias).
Additional prompt for combined input	Assess the symmetry of the funnel plot by examining the relationship between effect sizes and standard errors, where each point represents a trial. Supplement visual input with numerical input by analyzing the underlying data, including the distribution of effect sizes across different standard error levels, to provide a comprehensive evaluation of publication bias.

### 2.5 Evaluation Metrics

A false positive was recorded when the detection tool indicated the presence of PB in a dataset where no PB was introduced (i.e., m=0). A false negative occurred when the tool failed to detect PB in a dataset where bias was deliberately introduced (i.e., m > 0). To evaluate the performance of GPT‐4o and LIama 3.2 Vision in detecting PB using the simulated data, we used the following metrics:

$$\text{Sensitivity}\;(\text{Recall})=\frac{\text{TP}}{\text{TP}+\text{FN}};$$

$$\text{Specificity}\;=\frac{\text{TN}}{\text{TN}+\text{FP}};$$

$$\text{Positive Predictive Value}\;(\text{PPV})=\frac{\text{TP}}{\text{TP}+\text{FP}};$$

$$\text{Negative Predictive Value}\;(\text{NPV})=\frac{\text{TN}}{\text{TN}+\text{FN}};$$

$$F1\;\text{Score}=\frac{2\cdot \text{PPV}\cdot \text{Sensitivity}}{\text{PPV}+\text{Sensitivity}}=\frac{2\text{TP}}{2\text{TP}+\text{FP}+\text{FN}};$$

$$\text{Consistency Rate}\;(\text{Accuracy})=\frac{\text{TP}+\text{TN}}{\text{TP}+\text{TN}+\text{FP}+\text{FN}}.$$

To better illustrate the application of the AI tool on real‐world data, we included a case study as an example.

To assess the performance of our LLM‐based approach in detecting PB, we conducted a simulation study as a benchmark comparison with traditional methods. Egger's test [15], Begg's ranked test [30], and trim‐and‐fill method [31] were used to assess PB alongside our LLM‐based tool. We examined differences in inference and visual interpretation across methods, highlighting cases where the LLM approach provided additional insights or diverged from traditional metrics.

## 3 Results

### 3.1 Overall Performance of LLMs

The evaluation metrics for the two models are presented in Table 2. Each cell contains the mean across all scenarios and replications for a given model and input type, along with the corresponding confidence interval (CI). For GPT‐4o, there was no significant difference in predictive performance using the funnel plots input and combining it with statistical data, with F1 scores of 0.62 (95% CI: [0.61, 0.63]) versus 0.55 (95% CI: [0.54, 0.57]). The results for PPV (0.77 vs. 0.78), NPV (0.26 vs. 0.27), sensitivity (0.40 vs. 0.43), and specificity (0.64 vs. 0.64) were also similar. Similarly, LIama 3.2 Vision also displayed comparable predictive performance when utilizing both types of inputs (F1 scores = 0.62 with 95% CI 0.61–0.63 vs. 0.55 with 95% CI 0.54–0.57). The additional metrics, includingPPV (0.75 vs. 0.75), NPV (0.25 vs. 0.25), sensitivity (0.53 vs. 0.48), and specificity (0.48 vs. 0.52), also remained similar (Table 2). For the difference between the models, GPT‐4o generally demonstrated higher PPV, NPV, and specificity than Llama 3.2 Vision, but at the cost of lower sensitivity and, in some cases, a reduced F1 score. The details of subgroup analyses were presented in Supporting Information S1: Tables 1–4.

**Table 2:** Overall performance of LLMs in detection PB.

Model	Type of input	PPV	NPV	Sensitivity	Specificity	F1 Score
GPT‐4o	Visual input	0.77 (0.76, 0.78)	0.26 (0.25, 0.27)	0.40 (0.39, 0.41)	0.64 (0.63, 0.65)	0.62 (0.61, 0.63)
	Combined input	0.78 (0.77, 0.79)	0.27 (0.26, 0.28)	0.43 (0.42, 0.44)	0.64 (0.63, 0.65)	0.55 (0.54, 0.57)
Llama 3.2 Vision	Visual input	0.75 (0.74, 0.76)	0.25 (0.24, 0.26)	0.53 (0.52, 0.54)	0.48 (0.46, 0.49)	0.62 (0.61, 0.63)
	Combined input	0.75 (0.74, 0.76)	0.25 (0.24, 0.26)	0.48 (0.47, 0.49)	0.52 (0.51, 0.54)	0.59 (0.57, 0.60)

*Note:* The values in parentheses are 95% confidence intervals.

Abbreviations: NPV, negative predictive value; PPV, positive predictive value.

### 3.2 Trends in Subgroups Based on the Number of Studies

#### 3.2.1 Primary Analysis Results: Visual Inputs Only

We first presented the evaluation results based on the number of total studies within a meta‐analysis to explore whether the number of studies influences the performance of LLMs in detecting PB.

**Figure 1:**
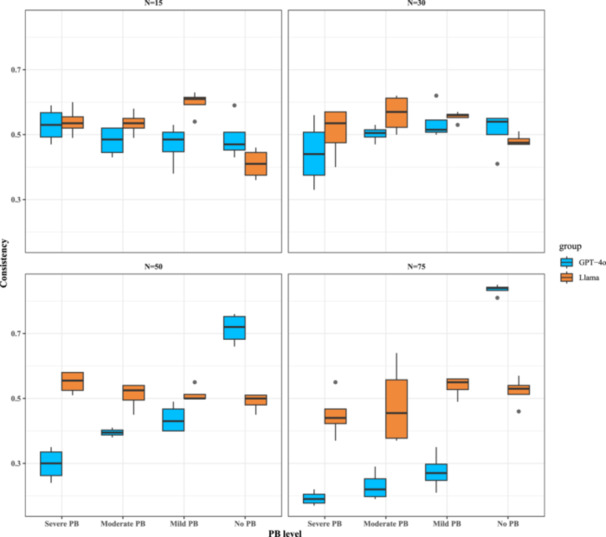
Simulation results of consistency rates for the primary analysis (visual inputs only) based on the number of studies. Each box represents the interquartile range (IQR) from Q1 (25th percentile) to Q3 (75th percentile), with the median (50th percentile) shown as a horizontal line. Whiskers extend to 1.5 times the IQR, while outliers beyond this range appear as individual points.

The results are summarized using box plots, as shown in Figure 1. Each box represents the estimated consistency rates obtained under the corresponding setting. The consistency rates reported in the following results sections all correspond to the solid line within each box. The accompanying 95% CIs were derived from 100 replications in our simulation study.

**Under the setting of no PB** (the rightmost boxes within each subplot), Llama 3.2 Vision (boxes in orange) remains stable across study groups, with consistency rates ranging from 0.41 (95% CI: 0.36–0.46) for 15 studies and 0.53 (95% CI: 0.46–0.57) for 75 studies. Based on the pattern, the performance of Llama was not affected by the total number of studies. We also presented the detailed results on more granular settings on different levels of between‐study heterogeneity and non‐publishing patterns in Supporting Information S1: Figure 1 (left panel), where the consistency rates of most subgroups were around 0.5.

Under the same no PB setting, GPT‐4o's consistency rate improved with more studies (blue boxes, rightmost in each sub‐figure). It increased from 0.47 (95% CI: 0.43–0.59) at 15 studies to 0.54 (95% CI: 0.41–0.55) at 30 studies, 0.72 (95% CI: 0.66–0.76) at 45 studies, and 0.84 (95% CI: 0.81–0.85) at 75 studies (Figure 1). Based on the detailed results of the consistency rates of most subgroups, the consistency rates for most subgroups were approximately 0.75 when the number of studies is large, but dropped to around 0.5 for smaller study groups (Supporting Information S1: Figure 1's right panel). This highlights the positive impact of larger sample sizes on the detection of absence of PB.

**Under PB conditions** (the three leftmost settings within each subplot), Llama 3.2 exhibited stable performance across the total number of studies. Consistency rates remained similar regardless of PB severity. For example, for 15 studies, consistency ranged from 0.54 (95% CI: 0.49–0.58) (moderate PB) to 0.61 (95% CI: 0.54–0.63) (mild PB), while for 75 studies, it varied between 0.45 (95% CI: 0.37–0.55) (severe PB) and 0.55 (95% CI: 0.49–0.56) (mild PB) (Figure 1). Despite this stability, Llama 3.2's relatively low consistency suggests limited reliability, with no significant improvement as study numbers increase.

GPT‐4o showed a clear trend: consistency rates decrease as the number of studies increases, particularly under severe PB and moderate PB. For severe PB, consistency dropped from 0.53 (95% CI: 0.47–0.59) for 15 studies to 0.19 (95% CI: 0.17–0.22) for 75 studies. Similarly, for moderate PB, it declined from 0.48 (95% CI: 0.43–0.52) to 0.23 (95% CI: 0.19–0.29).

To summarize, GPT‐4o more readily identified cases with no PB in larger meta‐analyses, likely due to more stable effect estimates. However, in doing so, some sensitivity may be sacrificed to detect the presence of PB. By contrast, Llama 3.2 showed relatively consistent (though less dynamic) performance across all scenarios of PB presence, with minimal dependence on the number of studies.

#### 3.2.2 Secondary Analysis Results: Combined Visual Inputs and Statistical Inputs

**Figure 2:**
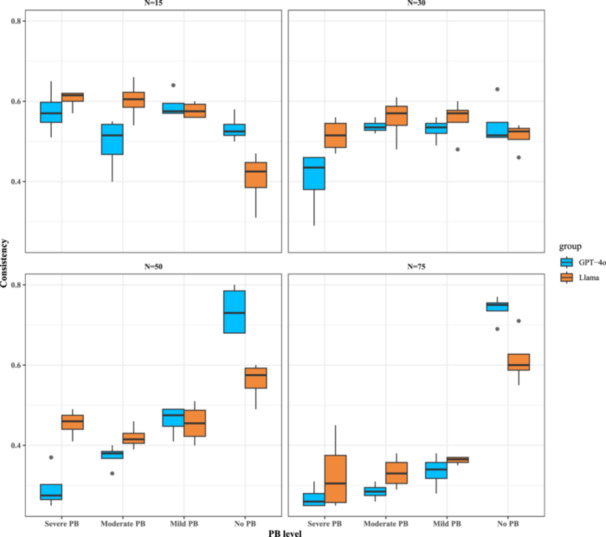
Simulation results of consistency rates for the secondary analysis (combined inputs) based on the number of studies. Each box represents the interquartile range (IQR) from Q1 (25th percentile) to Q3 (75th percentile), with the median (50th percentile) shown as a horizontal line. Whiskers extend to 1.5 times the IQR, while outliers beyond this range appear as individual points.

**In the no PB setting,** Llama 3.2 showed improved consistency with more studies, rising from 0.42 (95% CI: 0.31–0.47) at 15 studies to 0.60 (95% CI: 0.55–0.71) at 75 studies (Figure 2), suggesting a positive effect of combined inputs on accuracy. According to the detailed results on more granular settings on different levels of between‐study heterogeneity and non‐publishing patterns in Supporting Information S1: Figure 2 (left panel), the consistency rates of most subgroups were around 0.6.

Under the same no PB setting, GPT‐4o's consistency remained stable in the smaller studies group but increased as the number of studies grew. Specifically, for the groups with 15 studies and 30 studies, consistency remained relatively stable at 0.53 (95% CI: 0.50–0.58) and 0.52 (95% CI: 0.51–0.63), respectively. However, noticeable variations were observed in larger study groups. In groups with 50 and 75 studies, consistency increased significantly to 0.73 (95% CI: 0.68–0.80) and 0.74 (95% CI: 0.69–0.77) (Figure 2). According to the detailed results on more granular settings on different levels of between‐study heterogeneity and non‐publishing patterns in Supporting Information S1: Figure 2 (right panel), the consistency rates for most subgroups were approximately 0.75 when the number of studies is large, but dropped to around 0.5 for smaller study groups. The overall patterns in GPT‐4o remained like previous inference results based on plots, and there were no significant differences between the two kinds of inputs.

**In the presence of PB**, Llama remained relatively stable across different study group sizes regardless of PB severity (Figure 2). For instance, for 50 studies, the consistency rates were around 0.5, ranging from 0.42 (95% CI: 0.39–0.46) (moderate PB) to 0.46 (95% CI: 0.41–0.49) (severe PB). For the 30 studies, it varied between 0.52 (95% CI: 0.47–0.56) (severe PB) and 0.57 (95% CI: 0.48–0.61) (mild PB), but it dropped significantly to 0.29 (95% CI: 0.26–0.31) for 75 studies (Figure 2). The detailed results showed that the consistency rates of most subgroups were around 0.5 (Supporting Information S1: Figure 2's left panel).

For GPT‐4o, the pattern remained largely unchanged with the inference results based on plots (Figures 1 and 2). The consistency rates decreased as the number of studies increases, particularly under Severe PB and Moderate PB. For Severe PB, consistency dropped from 0.57 (95% CI: 0.51–0.65) at 15 studies to 0.26 (95% CI: 0.25–0.31) at 75 studies. Similarly, for Moderate PB, it declined from 0.52 (95% CI: 0.40–0.55) to 0.29 (95% CI: 0.26–0.31). The detailed results also showed a similar pattern: the consistency rates of most subgroups are around 0.5 (Supporting Information S1: Figure 2's right panel).

### 3.3 Trends in Subgroups Based on Heterogeneity and Non‐Reported Study Patterns

#### 3.3.1 Primary Analysis Results: Visual Inputs Only

To better understand the influence of heterogeneity and missing patterns, we visualized the results to highlight trends across different levels of between‐study heterogeneity and missing data patterns.

**Figure 3:**
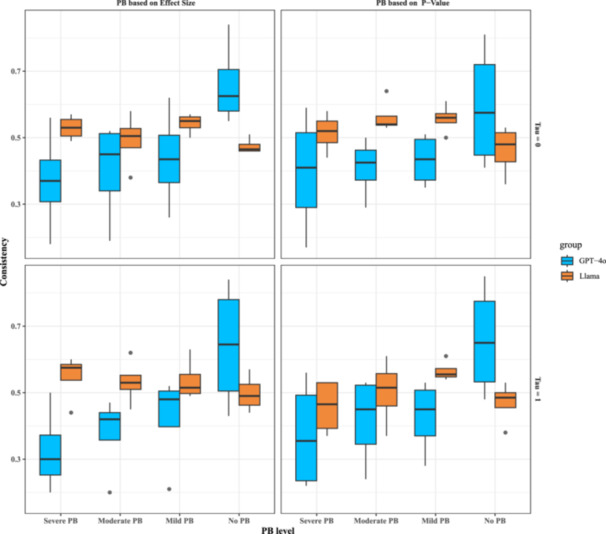
Simulation results of consistency rates for the primary analysis (visual inputs only) based on the missing patterns and heterogeneity. Each box represents the interquartile range (IQR) from Q1 (25th percentile) to Q3 (75th percentile), with the median (50th percentile) shown as a horizontal line. Whiskers extend to 1.5 times the IQR, while outliers beyond this range appear as individual points.

**For Llama, under the no PB setting,** the consistency across different missing patterns and heterogeneity remained relatively stable for Llama, fluctuating between 0.47 (95%CI: 0.46‐0.51) ($\tau^{2}$ = 0 and missing based on effect size) and 0.49 (95%CI: 0.38‐0.53) ($\tau^{2}$ = 1 and missing based on p‐value) (Figure 3). **In the presence of PB settings,** Llama 3.2 demonstrated relatively stable performance across different conditions of PB presence, missing patterns, and heterogeneity, as shown in Figure 3. The consistency rates remained within a similar range regardless of PB severity (Figure 3). For example, at $\tau^{2}$ = 0 and missing according to effect size, the Llama 3.2's consistency ranged from 0.50 (95% CI: 0.38–0.58) (moderate PB) to 0.55 (95% CI: 0.50–0.57) (mild PB) and reached 0.53 (95% CI: 0.49–0.57) for severe PB (Figure 2). The overall pattern shows the limited impact of missing patterns and heterogeneity on the Llama's inference (Supporting Information S1: Figure 2), regardless of the absence or presence of PB.

**For GPT‐4o in no PB groups**, the consistency rate is higher than Llama 3.2 for the corresponding setting. For example, it reached 0.63 (95% CI: 0.55–0.84) for $\tau^{2}$ = 0 and missing according to effect size, and 0.65 (95% CI: 0.48–0.85) for $\tau^{2}$ = 1 and missing based on p‐value (Figure 3). However, when examining the subgroup differences between missing patterns and heterogeneity, the similar CI indicated the differences were limited (Figure 3). For the presence of PB, GPT‐4o exhibited relatively low consistency compared to Llama. Specifically, in the severe PB groups, consistency was lower compared to other groups, dropping to 0.30 (95% CI: 0.20–0.50) when $\tau^{2}$ = 0 and studies were missing based on effect size. The detailed results on more granular settings (Supporting Information S1: Figure 1's left panel) also show the limited impact of missing patterns and heterogeneity on the GPT‐4o's inference, regardless of the absence or presence of PB. By the between‐study heterogeneity and missing patterns, GPT‐4o demonstrated relatively stable performance across different conditions.

#### 3.3.2 Secondary Analysis Results: Visual Inputs and Statistical Inputs

**Figure 4:**
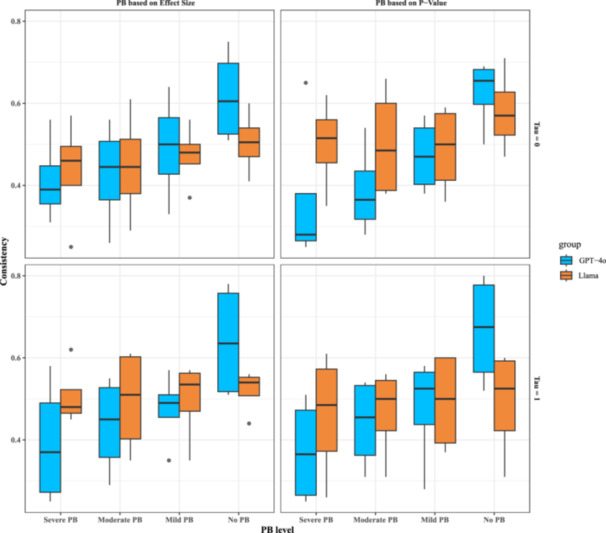
Simulation results of consistency rates for the secondary analysis (combined inputs) based on the missing patterns and heterogeneity. Each box represents the interquartile range (IQR) from Q1 (25th percentile) to Q3 (75th percentile), with the median (50th percentile) shown as a horizontal line. Whiskers extend to 1.5 times the IQR, while outliers beyond this range appear as individual points.

For the inference results based on combined inputs. In the no PB group, the results of Llama remained consistent with previous findings according to plot inputs, showing no significant deviations or improvements compared to the trends observed with visual inputs alone (Figures 3 and 4). For PB groups, the Llama's consistency rate also remained stable at around 0.5, with upper and lower quartiles, compared to the inference results based on plots (25th and 75th percentiles). The results aligned with our findings based on the number of studies.

The GPT‐4o showed higher consistency in detecting the absence of PB; the values fluctuated between 0.61 (95% CI: 0.51–0.75) ($\tau^{2}$ = 0 and missing based on effect size) and 0.68 (95% CI: 0.52–0.80) ($\tau^{2}$ = 1 and missing based on p‐value), which is also similar to the results based on plots (Figures 3 and 4). For PB groups, there was no difference found between different between‐study heterogeneity and missing patterns for consistency rates. The findings supported the results based on the plots. Overall, the combined information showed a limited effect on detection accuracy for LLMs.

### 3.4 Case Study of LLM in Real‐World Data

**Figure 5:**
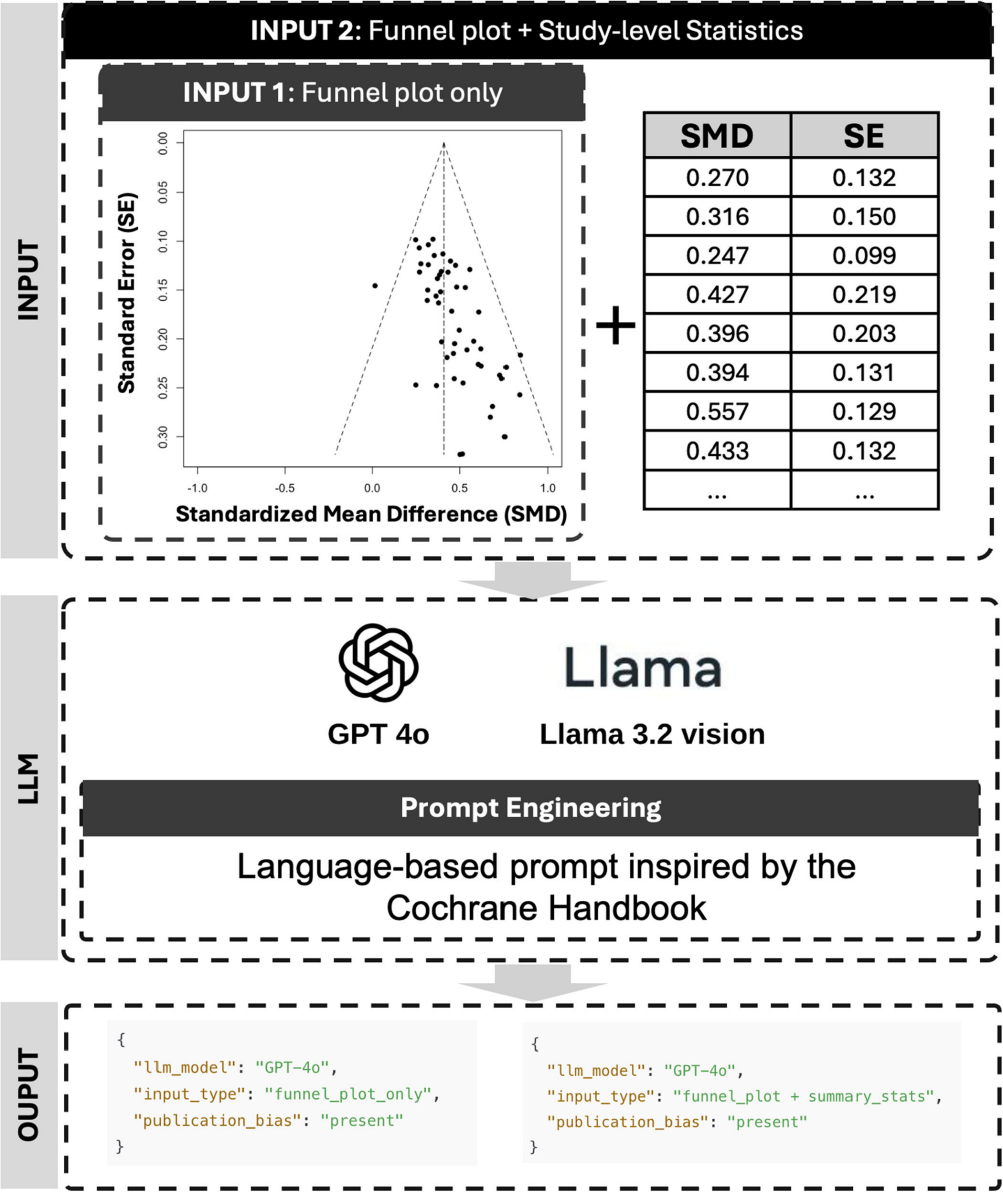
Workflow of the case study. The inputs to the LLMs include either a funnel plot image in PNG format alone or combined with JSON‐formatted study‐level summary statistics (SMD: standardized mean difference; SE: standard error). The output is a binary assessment indicating the presence or absence of publication bias (PB).

We conducted a case study to evaluate the performance of LLMs in identifying signals of PB from a real‐world meta‐analysis (Figure 5). Turner et al. [32] investigated the efficacy of 12 antidepressants reported in studies registered at the FDA. The effects are evaluated by the standardized mean difference (SMD) for antidepressants versus placebo, and larger SMDs are favored. The dataset contains both published results and results that were reported to the FDA but were not published.

As in the evaluation studies above, we tested two input settings for the LLM‐based analysis: (1) funnel plot only and (2) funnel plot combined with study‐level reported summary statistics. Using the LLM‐based workflow, GPT‐4o identified the presence of PB for both settings. Llama initially assessed the funnel plot alone as showing no evidence of PB. However, when the model was provided with both the funnel plot and the associated statistical information, Llama revised its assessment and identified the presence of potential PB. Egger's test yielded a *p*‐value < 0.001, further supporting the existence of PB. Together, these findings suggest consistent evidence of PB across traditional and LLM‐based approaches.

### 3.5 Comparison Between the Traditional Statistical Methods and LLMs

As shown in Supporting Information S1: Table 5, traditional PB tests exhibit very low power when a meta‐analysis includes only 15 studies (≈ 0.15, consistent with previous reports [25, 33]), whereas the LLM‐based methods reach about 0.50. Traditional tests, however, maintain a lower Type I error rate, indicating stronger control over false positives in small samples. When the number of studies increases to 30, the power of traditional tests rises to roughly 0.40, narrowing but not eliminating the performance gap. With 50 or 75 studies, the advantage of LLMs diminishes, as both approaches achieve higher power ( ≈ 0.70–0.80).

## 4 Discussion

Automation of systematic reviews seems inevitable, and LLMs can be a key driver to achieve this goal. In this study, we evaluated two state‐of‐the‐art multimodal LLMs, GPT‐4o and Llama 3.2 Vision, by simulating meta‐analyses under varying conditions of PB level, study number, and between‐study heterogeneity. By using funnel plots alone, the most commonly used graph‐based method for PB detection, and in combination with supplementary statistical data, we found that although GPT‐4o performs somewhat better at identifying the absence of PB in a larger number of studies, neither model reliably detects the presence of PB without additional fine‐tuning. Including statistical inputs did not significantly enhance performance, and between‐study heterogeneity or missing data patterns had only minimal impact on overall accuracy.

Our study offers several key advantages. Firstly, we have conducted comprehensive simulation studies by mimicking realistic settings. By varying levels of PB, the number of studies, missing patterns, and heterogeneity, the simulation covered a broad range of scenarios that meta‐analysts might encounter in practice, which provided enough evidence to support the robustness of our findings. Secondly, we compared two state‐of‐the‐art multimodal models. Current research on applying LLMs to meta‐analyses, especially for PB detection, remains sparse. Rigorous evaluations, like those presented in this study, are therefore crucial for informing users and guiding future model refinement. However, the performance of LLMs was limited based on our findings. With continued development and validation, LLMs could serve as invaluable aids to researchers and clinicians, streamlining evidence synthesis and increasing confidence in meta‐analytic conclusions. Thirdly, our evaluation goes beyond relying solely on funnel plots by also incorporating relevant statistical information into LLM‐based analyses. This integrative approach enhanced the robustness of findings by allowing the models to consider both visual patterns and numerical evidence. Lastly, we employed targeted prompt engineering to optimize how LLMs process and interpret both graphical and statistical data, further enhancing their capacity to detect subtle indications of bias.

Nevertheless, there are still many ways future research could be improved. First, only GPT‐4o and Llama 3.2 Vision were considered. Future research might include additional or fine‐tuned models to determine whether performance varies across architectures. Second, the lack of real‐world validation due to the complexities of PB mechanisms is hard to capture in real‐world evidence [34, 35]. Inverse PB [17, 34, 36] also brings a challenge to the current evidence synthesis practice as it involves special considerations. Future work should incorporate real‐world evidence, such as data from the FDA, to fine‐tune models, enabling them to better address challenges in clinical practice. FDA data, which includes both published and unpublished trial results, can provide valuable insights into the true extent of PB [37]. By leveraging such comprehensive datasets, models can be trained to recognize real‐world PB patterns more effectively. Our simulation design is built on predefined selection functions (based on p‐values or effect sizes), which, while grounded in the empirical literature, may not fully capture the adaptive, field‐specific, or sociological drivers of real‐world PB, such as journal policies, author incentives, or topic novelty. In particular: (1) Real publication processes may involve multi‐layered selection, which interact non‐linearly and may not map cleanly onto a simple threshold or probability gradient. (2) Endogenous PB may correlate with factors like study design, funding source, disease area, or perceived novelty, which were not modeled in our simulations. (3) The distribution of unpublished studies in reality is likely far from random and may include both extreme nonsignificant results and selectively omitted moderate ones, depending on field norms. Third, no fine‐tuning was conducted in our study. Fine‐tuning may benefit from training on labeled data to enhance PB detection capabilities. Future work should focus on developing and optimizing specialized LLMs for PB detection to better support evidence‐based research. An ideal model should go beyond providing a simple binary decision on the presence of PB. It should also identify and describe the specific patterns of missing studies in the funnel plot. The inclusion of additional descriptive statistical data has not significantly improved the performance, highlighting the limitations of the current approach. Future model training may benefit from incorporating the metrics bias detection methods (e.g., regression‐based metrics) to better capture complex PB mechanisms. Fourth, one design choice in our simulation was to draw within‐study standard errors from a Uniform(1,4) distribution. This range was selected to reflect the variability in study precision commonly observed in real‐world meta‐analyses, especially those including a mix of small and large studies. We recognize, however, that the average standard error under this setting is relatively high, which may not match every applied context. Future work could explore narrower intervals, empirically‐derived distributions from published meta‐analyses, or heteroskedastic structures where standard errors are correlated with effect sizes or sample sizes. Such extensions would allow for even closer alignment with the complexity of real‐world data. Although our study was not pre‐registered on a public platform (e.g., OSF), we have made all code, data, and the study protocol openly available in a time‐stamped repository. This supports transparency and reproducibility in line with open science and open‐source principles.

Future research can explore how artificial intelligence (AI) tools can be leveraged for the detection of PB beyond traditional statistical methods [38, 39]. Future studies can also explore interactive workflows where LLMs assist human reviewers in the assessment of publication bias, thereby improving overall efficiency and consistency. AI‐driven approaches could be used to assist in systematic analysis of trial registries (e.g., ClinicalTrials.gov, WHO ICTRP) to identify studies that have been conducted but remain unpublished. Additionally, AI tools with advanced text understanding capabilities could be utilized to scan and analyze non‐English publications, which are often excluded from systematic reviews and meta‐analyses, potentially leading to language‐related PB. By integrating machine learning and natural language processing (NLP), AI tools could enhance transparency in research dissemination and provide a more comprehensive assessment of PB.

## Author Contributions

**Xing Xing:** conceptualization, methodology, investigation, data curation, formal analysis, writing – original draft, writing – review and editing, visualization. **Lifeng Lin:** methodology, writing – review and editing. **Mohammad Hassan Murad:** writing – review and editing. **Jiayi Tong:** conceptualization,methodology, supervision, visualization, writing – original draft, writing – review and editing, project administration.

## Conflicts of Interest

The authors declare no conflicts of interest.

## Peer Review

The peer review history for this article is available at https://www.webofscience.com/api/gateway/wos/peer-review/10.1002/cesm.70047.

## Supporting information


**Supplementary Figure 1:** Consistency heatmap of LLMs using the visual input. **Supplementary Figure 2:** Consistency heatmap of LLMs using the combined input. **Supplementary Table 1:** The agreement rate across different level of PB levels using the visual input. **Supplementary Table 2:** The agreement rate across different level of PB levels using the combined input. **Supplementary Table 3:** The agreement rate across different missing patterns and tau using the visual input. **Supplementary Table 4:** The agreement rate across different missing patterns and tau using the combined input. **Supplementary Table 5:** Type I error and power of comprehensive methods to detect publication bias.

## Data Availability

All statistical code and data used for implementing the methods are publicly available at https://osf.io/e2quz/.
