# Supplementary material for: Leveraging AI for Meta‐Analysis: Evaluating LLMs in Detecting Publication Bias for Next‐Generation Evidence Synthesis
Source: Cochrane Evid Synth Methods. 2025 Sep 18;3(5):e70047. doi: 10.1002/cesm.70047 (PMC12463026; doi:10.1002/cesm.70047)
Supplement: Supplementary file 1 — Supplementary Figure 1: Consistency heatmap of LLMs using the visual input. Supplementary Figure 2: Consistency heatmap of LLMs using the combined input. Supplementary Table 1: The agreement rate across different level of PB levels using the visual input. Supplementary Table 2: The agreement rate across different level of PB levels using the combined input. Supplementary Table 3: The agreement rate across different missing patterns and tau using the visual input. Supplementary Table 4: The agreement rate across different missing patterns and tau using the combined input. Supplementary Table 5: Type I error and power of comprehensive methods to detect publication bias. [file CESM-3-e70047-s001.docx]

**Supplementary File of “Leveraging AI for Meta-Analysis: Evaluating LLMs in Detecting Publication Bias for Next-Generation Evidence Synthesis”**

Supplementary Figure 1. Consistency heatmap of LLMs using the visual input

Supplementary Figure 2. Consistency heatmap of LLMs using the combined input

Supplementary Table 1: The agreement rate across different level of PB levels using the visual input

Supplementary Table 2: The agreement rate across different level of PB levels using the combined input

Supplementary Table 3: The agreement rate across different missing patterns and tau using the visual input

Supplementary Table 4: The agreement rate across different missing patterns and tau using the combined input

Supplementary Table 5: Type I error and power of comprehensive methods to detect publication bias

Supplementary Figure 1. Consistency heatmap of LLMs using the visual input

****Supplementary Figure 2. Consistency heatmap of LLMs using the combined input

| Number of studies | PB level | Group | Agreement Median | Lower CI | Upper CI |
| --- | --- | --- | --- | --- | --- |
| 15 | Mild PB | GPT | 0.49 | 0.38 | 0.53 |
| 15 | Mild PB | Llama | 0.61 | 0.54 | 0.63 |
| 15 | Moderate PB | GPT | 0.49 | 0.43 | 0.52 |
| 15 | Moderate PB | Llama | 0.54 | 0.49 | 0.58 |
| 15 | No PB | GPT | 0.47 | 0.43 | 0.59 |
| 15 | No PB | Llama | 0.41 | 0.36 | 0.46 |
| 15 | Severe PB | GPT | 0.53 | 0.47 | 0.59 |
| 15 | Severe PB | Llama | 0.54 | 0.49 | 0.6 |
| 30 | Mild PB | GPT | 0.52 | 0.5 | 0.62 |
| 30 | Mild PB | Llama | 0.56 | 0.53 | 0.57 |
| 30 | Moderate PB | GPT | 0.51 | 0.47 | 0.53 |
| 30 | Moderate PB | Llama | 0.57 | 0.5 | 0.62 |
| 30 | No PB | GPT | 0.54 | 0.41 | 0.55 |
| 30 | No PB | Llama | 0.48 | 0.47 | 0.51 |
| 30 | Severe PB | GPT | 0.44 | 0.33 | 0.56 |
| 30 | Severe PB | Llama | 0.54 | 0.4 | 0.57 |
| 50 | Mild PB | GPT | 0.43 | 0.4 | 0.49 |
| 50 | Mild PB | Llama | 0.50 | 0.5 | 0.55 |
| 50 | Moderate PB | GPT | 0.40 | 0.38 | 0.41 |
| 50 | Moderate PB | Llama | 0.53 | 0.45 | 0.54 |
| 50 | No PB | GPT | 0.72 | 0.66 | 0.76 |
| 50 | No PB | Llama | 0.50 | 0.45 | 0.51 |
| 50 | Severe PB | GPT | 0.30 | 0.24 | 0.35 |
| 50 | Severe PB | Llama | 0.56 | 0.51 | 0.58 |
| 75 | Mild PB | GPT | 0.27 | 0.21 | 0.35 |
| 75 | Mild PB | Llama | 0.55 | 0.49 | 0.56 |
| 75 | Moderate PB | GPT | 0.22 | 0.19 | 0.29 |
| 75 | Moderate PB | Llama | 0.46 | 0.37 | 0.64 |
| 75 | No PB | GPT | 0.84 | 0.81 | 0.85 |
| 75 | No PB | Llama | 0.53 | 0.46 | 0.57 |
| 75 | Severe PB | GPT | 0.19 | 0.17 | 0.22 |
| 75 | Severe PB | Llama | 0.44 | 0.37 | 0.55 |
|  | | | | | |

Supplementary Table 1: The agreement rate across different level of PB levels using the visual input

Supplementary Table 2: The agreement rate across different level of PB levels using the combined input

| Number of studies | PB level | Group | Agreement Median | Lower CI | Upper CI |
| --- | --- | --- | --- | --- | --- |
| 15 | Mild PB | Llama | 0.58 | 0.56 | 0.6 |
| 15 | Mild PB | GPT | 0.58 | 0.57 | 0.64 |
| 15 | Moderate PB | Llama | 0.61 | 0.54 | 0.66 |
| 15 | Moderate PB | GPT | 0.52 | 0.4 | 0.55 |
| 15 | No PB | Llama | 0.43 | 0.31 | 0.47 |
| 15 | No PB | GPT | 0.53 | 0.5 | 0.58 |
| 15 | Severe PB | Llama | 0.62 | 0.57 | 0.62 |
| 15 | Severe PB | GPT | 0.57 | 0.51 | 0.65 |
| 30 | Mild PB | Llama | 0.57 | 0.48 | 0.6 |
| 30 | Mild PB | GPT | 0.54 | 0.49 | 0.56 |
| 30 | Moderate PB | Llama | 0.57 | 0.48 | 0.61 |
| 30 | Moderate PB | GPT | 0.54 | 0.52 | 0.56 |
| 30 | No PB | Llama | 0.53 | 0.46 | 0.54 |
| 30 | No PB | GPT | 0.52 | 0.51 | 0.63 |
| 30 | Severe PB | Llama | 0.52 | 0.47 | 0.56 |
| 30 | Severe PB | GPT | 0.44 | 0.29 | 0.46 |
| 50 | Mild PB | Llama | 0.46 | 0.4 | 0.51 |
| 50 | Mild PB | GPT | 0.48 | 0.41 | 0.49 |
| 50 | Moderate PB | Llama | 0.42 | 0.39 | 0.46 |
| 50 | Moderate PB | GPT | 0.38 | 0.33 | 0.4 |
| 50 | No PB | Llama | 0.58 | 0.49 | 0.6 |
| 50 | No PB | GPT | 0.73 | 0.68 | 0.8 |
| 50 | Severe PB | Llama | 0.46 | 0.41 | 0.49 |
| 50 | Severe PB | GPT | 0.28 | 0.25 | 0.37 |
| 75 | Mild PB | Llama | 0.37 | 0.35 | 0.37 |
| 75 | Mild PB | GPT | 0.34 | 0.28 | 0.38 |
| 75 | Moderate PB | Llama | 0.33 | 0.29 | 0.38 |
| 75 | Moderate PB | GPT | 0.29 | 0.26 | 0.31 |
| 75 | No PB | Llama | 0.60 | 0.55 | 0.71 |
| 75 | No PB | GPT | 0.75 | 0.69 | 0.77 |
| 75 | Severe PB | Llama | 0.31 | 0.25 | 0.45 |
| 75 | Severe PB | GPT | 0.26 | 0.25 | 0.31 |
|  | | | | | |

Supplementary Table 3: The agreement rate across different missing patterns and tau using the visual input

| Scenario | Tau | PB level | Group | Agreement Median | Lower CI | Upper CI |
| --- | --- | --- | --- | --- | --- | --- |
| Missing based on Effect Size | 0 | Mild PB | GPT | 0.44 | 0.26 | 0.62 |
| Missing based on Effect Size | 0 | Mild PB | Llama | 0.55 | 0.5 | 0.57 |
| Missing based on Effect Size | 0 | Moderate PB | GPT | 0.45 | 0.19 | 0.52 |
| Missing based on Effect Size | 0 | Moderate PB | Llama | 0.51 | 0.38 | 0.58 |
| Missing based on Effect Size | 0 | No PB | GPT | 0.63 | 0.55 | 0.84 |
| Missing based on Effect Size | 0 | No PB | Llama | 0.47 | 0.46 | 0.51 |
| Missing based on Effect Size | 0 | Severe PB | GPT | 0.37 | 0.18 | 0.56 |
| Missing based on Effect Size | 0 | Severe PB | Llama | 0.53 | 0.49 | 0.57 |
| Missing based on Effect Size | 1 | Mild PB | GPT | 0.48 | 0.21 | 0.52 |
| Missing based on Effect Size | 1 | Mild PB | Llama | 0.52 | 0.49 | 0.63 |
| Missing based on Effect Size | 1 | Moderate PB | GPT | 0.42 | 0.2 | 0.47 |
| Missing based on Effect Size | 1 | Moderate PB | Llama | 0.53 | 0.45 | 0.62 |
| Missing based on Effect Size | 1 | No PB | GPT | 0.65 | 0.43 | 0.84 |
| Missing based on Effect Size | 1 | No PB | Llama | 0.49 | 0.44 | 0.57 |
| Missing based on Effect Size | 1 | Severe PB | GPT | 0.30 | 0.2 | 0.5 |
| Missing based on Effect Size | 1 | Severe PB | Llama | 0.58 | 0.44 | 0.6 |
| Missing based on P-value | 0 | Mild PB | GPT | 0.44 | 0.35 | 0.51 |
| Missing based on P-value | 0 | Mild PB | Llama | 0.56 | 0.5 | 0.61 |
| Missing based on P-value | 0 | Moderate PB | GPT | 0.43 | 0.29 | 0.5 |
| Missing based on P-value | 0 | Moderate PB | Llama | 0.54 | 0.53 | 0.64 |
| Missing based on P-value | 0 | No PB | GPT | 0.58 | 0.41 | 0.81 |
| Missing based on P-value | 0 | No PB | Llama | 0.48 | 0.36 | 0.53 |
| Missing based on P-value | 0 | Severe PB | GPT | 0.41 | 0.17 | 0.59 |
| Missing based on P-value | 0 | Severe PB | Llama | 0.52 | 0.44 | 0.58 |
| Missing based on P-value | 1 | Mild PB | GPT | 0.45 | 0.28 | 0.53 |
| Missing based on P-value | 1 | Mild PB | Llama | 0.56 | 0.54 | 0.61 |
| Missing based on P-value | 1 | Moderate PB | GPT | 0.45 | 0.24 | 0.53 |
| Missing based on P-value | 1 | Moderate PB | Llama | 0.52 | 0.37 | 0.61 |
| Missing based on P-value | 1 | No PB | GPT | 0.65 | 0.48 | 0.85 |
| Missing based on P-value | 1 | No PB | Llama | 0.49 | 0.38 | 0.53 |
| Missing based on P-value | 1 | Severe PB | GPT | 0.36 | 0.22 | 0.56 |
| Missing based on P-value | 1 | Severe PB | Llama | 0.47 | 0.37 | 0.53 |
|  |  |  |  |  |  |  |

Supplementary Table 4: The agreement rate across different missing patterns and tau using the combined input

| Scenario | Tau | PB level | Group | Agreement Median | Lower CI | Upper CI |
| --- | --- | --- | --- | --- | --- | --- |
| Missing based on Effect Size | 0 | Mild PB | Llama | 0.48 | 0.37 | 0.56 |
| Missing based on Effect Size | 0 | Mild PB | GPT | 0.50 | 0.33 | 0.64 |
| Missing based on Effect Size | 0 | Moderate PB | Llama | 0.45 | 0.29 | 0.61 |
| Missing based on Effect Size | 0 | Moderate PB | GPT | 0.45 | 0.26 | 0.56 |
| Missing based on Effect Size | 0 | No PB | Llama | 0.51 | 0.41 | 0.6 |
| Missing based on Effect Size | 0 | No PB | GPT | 0.61 | 0.51 | 0.75 |
| Missing based on Effect Size | 0 | Severe PB | Llama | 0.46 | 0.25 | 0.57 |
| Missing based on Effect Size | 0 | Severe PB | GPT | 0.39 | 0.31 | 0.56 |
| Missing based on Effect Size | 1 | Mild PB | Llama | 0.54 | 0.35 | 0.57 |
| Missing based on Effect Size | 1 | Mild PB | GPT | 0.49 | 0.35 | 0.57 |
| Missing based on Effect Size | 1 | Moderate PB | Llama | 0.51 | 0.35 | 0.61 |
| Missing based on Effect Size | 1 | Moderate PB | GPT | 0.45 | 0.29 | 0.55 |
| Missing based on Effect Size | 1 | No PB | Llama | 0.54 | 0.44 | 0.56 |
| Missing based on Effect Size | 1 | No PB | GPT | 0.64 | 0.51 | 0.78 |
| Missing based on Effect Size | 1 | Severe PB | Llama | 0.48 | 0.45 | 0.62 |
| Missing based on Effect Size | 1 | Severe PB | GPT | 0.37 | 0.25 | 0.58 |
| Missing based on P-value | 0 | Mild PB | Llama | 0.50 | 0.36 | 0.59 |
| Missing based on P-value | 0 | Mild PB | GPT | 0.47 | 0.38 | 0.57 |
| Missing based on P-value | 0 | Moderate PB | Llama | 0.49 | 0.38 | 0.66 |
| Missing based on P-value | 0 | Moderate PB | GPT | 0.37 | 0.28 | 0.54 |
| Missing based on P-value | 0 | No PB | Llama | 0.57 | 0.47 | 0.71 |
| Missing based on P-value | 0 | No PB | GPT | 0.66 | 0.5 | 0.69 |
| Missing based on P-value | 0 | Severe PB | Llama | 0.52 | 0.35 | 0.62 |
| Missing based on P-value | 0 | Severe PB | GPT | 0.28 | 0.25 | 0.65 |
| Missing based on P-value | 1 | Mild PB | Llama | 0.50 | 0.37 | 0.6 |
| Missing based on P-value | 1 | Mild PB | GPT | 0.53 | 0.28 | 0.58 |
| Missing based on P-value | 1 | Moderate PB | Llama | 0.50 | 0.31 | 0.56 |
| Missing based on P-value | 1 | Moderate PB | GPT | 0.46 | 0.31 | 0.54 |
| Missing based on P-value | 1 | No PB | Llama | 0.53 | 0.31 | 0.6 |
| Missing based on P-value | 1 | No PB | GPT | 0.68 | 0.52 | 0.8 |
| Missing based on P-value | 1 | Severe PB | Llama | 0.49 | 0.26 | 0.61 |
| Missing based on P-value | 1 | Severe PB | GPT | 0.37 | 0.25 | 0.51 |
|  |  |  |  |  |  |  |

Supplementary Table 5: Type I error and power of comprehensive methods to detect publication bias

|  |  |  |  |  |  |  |  |  |  |  |  |  |  |  |  |  |  |  |  |  |
| --- | --- | --- | --- | --- | --- | --- | --- | --- | --- | --- | --- | --- | --- | --- | --- | --- | --- | --- | --- | --- |
|  |  | **Missing based on effect size** | | | | | | | | |  | **Missing based on P-value** | | | | | | | | |
|  |  | **Tau = 0** | | | |  | **Tau = 1** | | | |  | **Tau = 0** | | | |  | **Tau = 1** | | | |
| **Test** |  | **m=0** | **n/10** | **n/5** | **n/3** |  | **m=0** | **n/10** | **n/5** | **n/3** |  | **m=0** | **n/10** | **n/5** | **n/3** |  | **m=0** | **n/10** | **n/5** | **n/3** |
| ***n = 15*** |  |  |  |  |  |  |  |  |  |  |  |  |  |  |  |  |  |  |  |  |
| Egger's Reg |  | 8 | 17 | 22 | 26 |  | 11 | 15 | 19 | 26 |  | 12 | 15 | 17 | 32 |  | 8 | 9 | 10 | 18 |
| Rank: two sided |  | 5 | 13 | 13 | 23 |  | 8 | 10 | 12 | 21 |  | 8 | 11 | 9 | 24 |  | 6 | 8 | 14 | 15 |
| Trim&Fill |  | 7 | 16 | 30 | 39 |  | 11 | 17 | 34 | 47 |  | 6 | 10 | 11 | 23 |  | 5 | 4 | 11 | 16 |
| ***n = 30*** |  |  |  |  |  |  |  |  |  |  |  |  |  |  |  |  |  |  |  |  |
| Egger's Reg |  | 8 | 23 | 44 | 46 |  | 7 | 18 | 41 | 47 |  | 14 | 13 | 27 | 37 |  | 10 | 12 | 19 | 20 |
| Rank: two sided |  | 3 | 17 | 31 | 36 |  | 5 | 11 | 33 | 36 |  | 7 | 15 | 20 | 35 |  | 5 | 5 | 17 | 19 |
| Trim&Fill |  | 7 | 43 | 67 | 82 |  | 3 | 42 | 68 | 89 |  | 8 | 7 | 18 | 15 |  | 9 | 0 | 13 | 3 |
| ***n = 50*** |  |  |  |  |  |  |  |  |  |  |  |  |  |  |  |  |  |  |  |  |
| Egger's Reg |  | 11 | 29 | 61 | 82 |  | 9 | 33 | 49 | 61 |  | 9 | 22 | 31 | 59 |  | 14 | 14 | 25 | 46 |
| Rank: two sided |  | 9 | 21 | 52 | 74 |  | 4 | 29 | 42 | 66 |  | 6 | 17 | 28 | 54 |  | 8 | 9 | 20 | 40 |
| Trim&Fill |  | 4 | 66 | 93 | 99 |  | 3 | 73 | 94 | 99 |  | 9 | 7 | 16 | 23 |  | 9 | 6 | 13 | 12 |
| ***n = 75*** |  |  |  |  |  |  |  |  |  |  |  |  |  |  |  |  |  |  |  |  |
| Egger's Reg |  | 6 | 33 | 75 | 95 |  | 7 | 24 | 62 | 79 |  | 11 | 24 | 44 | 71 |  | 13 | 23 | 28 | 52 |
| Rank: two sided |  | 5 | 30 | 64 | 90 |  | 9 | 15 | 51 | 76 |  | 10 | 21 | 45 | 66 |  | 8 | 16 | 24 | 53 |
| Trim&Fill |  | 8 | 82 | 97 | 100 |  | 6 | 85 | 99 | 99 |  | 24 | 8 | 31 | 18 |  | 17 | 13 | 12 | 17 |
|  |  |  |  |  |  |  |  |  |  |  |  |  |  |  |  |  |  |  |  |  |
